# Community perspectives on randomisation and fairness in a cluster randomised controlled trial in Zambia

**DOI:** 10.1186/s12910-019-0421-7

**Published:** 2019-12-21

**Authors:** Maureen Mupeta Kombe, Joseph Mumba Zulu, Charles Michelo, Ingvild F. Sandøy

**Affiliations:** 1 School of Public Health, University of Zambia, Ridgeway Campus, P.O Box 50110, Lusaka, Zambia; 2 Centre for Intervention Science in Maternal and Child Health (CISMAC), Centre for International Health, Department of Global Public Health and Primary Care, University of Bergen, Bergen, Norway

**Keywords:** Perception, Fairness, Understanding, Randomisation, “Lottery”, Cluster randomised trial

## Abstract

**Background:**

One important ethical issue in randomised controlled trials (RCTs) is randomisation. Relatively little is known about how participating individuals and communities understand and perceive central aspects of randomisation such as equality, fairness, transparency and accountability in community-based trials. The aim of this study was to understand and explore study communities’ perspectives of the randomisation process in a cluster RCT in rural Zambia studying the effectiveness of different support packages for adolescent girls on early childbearing.

**Methods:**

In this explorative study, in-depth semi-structured interviews were carried out in 2018 with 14 individuals who took part in the randomisation process of the Research Initiative to Support the Empowerment of Girls (RISE) project in 2016 and two traditional leaders. Two of the districts where the trial is implemented were purposively selected. Interviews were audio recorded and fully transcribed. Data were analysed by coding and describing emergent themes.

**Results:**

The understanding of the randomisation process varied. Some respondents understood that randomisation was conducted for research purposes, but most of them did not. They had trouble distinguishing research and aid. Generally, respondents perceived the randomisation process as transparent and fair. However, people thought that there should not have been a “lottery” because they wanted all schools to receive equal or balanced benefits of the interventions.

**Conclusions:**

Randomisation was misunderstood by most respondents. Perceived procedural fairness was easier to realize than substantive fairness. Researchers working on Cluster Randomised Controlled Trials (CRCTs) should consider carefully how to explain randomisation.

## Background

Randomised Controlled Trials (RCTs) are an important tool and increasingly being conducted to measure the effectiveness of health interventions, also in low and middle income countries (LMICs). They are considered the strongest research design for evaluating the effects of health interventions and are an important tool for evaluating social policies [3, 7, 8, 18, 22, 33, 35, 37, 38]. Some of the reasons are that randomisation reduces bias, and facilitates blinding (masking) of the identity of treatments from researchers, participants and assessors [1, 9, 10, 11, 15, 16, 28, 35].

Cluster Randomised Controlled Trials (CRCTs) are experiments in which intact social units or clusters of individuals are randomly allocated to intervention groups [1, 9, 15]. The use of clusters rather than independent individuals as randomisation units is relevant when the interventions that are being studied address groups or communities. Some examples of intact social units include communities, hospitals, workplaces, schools, medical practices, and bars [27]. The word “controlled” refers to the use of a control group, with similar characteristics as those who receive the interventions, that provides information on the prevalence and incidence of the outcomes of interest in the absence of the studied interventions. Randomisation is a process by which allocation of participants to a study group is done by chance [1, 10, 11, 15, 16, 17, 28]. When the randomisation is successful, that is cannot be predicted in advance by anyone and is completely impartial, the characteristics of the participants in the different arms will be similar, and any differences will be due to chance, not systematic biases [7, 11, 15, 16, 17, 18, 28]. A successful randomisation is a prerequisite for the internal validity of a trial. If the randomisation is flawed, the findings of the study may be confounded and the effectiveness of the interventions cannot be accurately measured [12, p. 38, 13].

Although a successful randomisation is essential in a RCT, it may be difficult to explain randomisation to potential participants in a way that makes them fully understand why it is necessary and what it implies [1, 7, 11, 22]. Appreciating that the participants have an equal chance of ending up in the treatment groups and that the allocation is impartial, may be particularly important if the participants perceive one of the treatments/interventions to be more beneficial than the other [15, 16, 22, 25, 26, 29, 31, 39, 41]. If the participants do not appreciate the rationale for the randomisation, or if they suspect that the process of treatment allocation could be manipulated, they may be less compliant to the treatment they are allocated to and more prone to withdraw from the study. In a cluster RCT, one consequence could be that whole clusters withdraw, and considering that the number of randomisation units in such trials often is low, this may seriously affect the power of the study.

In order to avoid suspicions that the randomisation could be unfair, that is biased because the investigators consciously or unconsciously favored some of the clusters, several trials of public health interventions have conducted the randomisation as part of a public ceremony [24]. The aims of such public randomisation ceremonies are frequently to increase awareness about the trial in the community, to create an understanding of the rational for the randomisation and to carry it out in a transparent manner which is easy to understand and will convince key stakeholders and participants that it was free from manipulation [11 (p. 100), 18, 19]. However, we are not aware of any studies that have explored how the individuals who are present at such public ceremonies and their communities understand and perceive the randomisation after the ceremonies are conducted. Do the ceremonies convince them that the randomisation is fair, transparent and that the people conducting the ceremonies are accountable? We had the opportunity to look into this in relation to the CRCT called the “Research Initiative to Support the Empowerment of Girls” (RISE) project in Zambia [27]. To address these questions, we conducted qualitative interviews with people who had participated in the randomisation ceremonies of the RISE study and with two traditional leaders. The study was expected to provide useful information on how to explain to research participants what the objective of randomisation is in community-based cluster randomised controlled trials.

### Background to the RISE project

In Zambia, 35% of young girls in rural areas, have given birth by the age of 18 years. Pregnancy rates are particularly high among girls who have dropped out of school [4, 5]. Poverty, low enrolment in secondary school, myths and community norms are some of the contributory factors to early childbearing [20, 23]. Adolescent pregnancies pose a risk to the young mothers and their babies [21, 36, 40]. Encouraging girls to get more education and postpone pregnancy and marriage until adulthood will promote healthier and more prosperous lives for girls and their communities. The RISE project tests the effectiveness of providing economic support alone or in combination with community dialogue on adolescent childbearing, early marriage and school dropout. The study is implemented in rural areas of 12 study districts in 2 provinces of Zambia. The trial has three study arms and 157 schools in total: Group 1, the control group (31 schools) received material support comprising of books, and pencils/pens. Group 2, the economic support arm comprising 63 schools received materials and economic support; and group 3, the combined intervention arm with 63 schools, received materials, economic support and community dialogue. The support packages were provided from September 2016 to November 2018 [27].

Girls in grade 7 (in 2016) and their parents/guardians were recruited between March and July 2016 after giving their assent/consent. The randomisation in the RISE trial was conducted in July 2016 and stratified by districts to ensure that all districts were represented in each arm. There was community engagement before and after the RISE randomisation ceremonies. Chiefs, representatives from the District Educational Board Secretary (DEBS) offices, headmasters and Parent Teacher Association (PTA) chairpersons of the trial schools in the two study districts that were included in each ceremony, were invited to be present at the ceremonies. Chiefs or their representatives assisted in drawing tickets from three small boxes [27]. For better understanding of the randomisation process by community members, randomisation was explained as an analogy to “lottery” and the randomisation process as a “ceremony”.

## Methods

This study was done within a CRCT to explore perceptions and experiences among community members related to the public randomisation process in the RISE project, in particular whether they perceived it as fair and transparent.

### Study design

Our study was qualitative explorative in nature. In this study, perceptions and experiences of participants regarding the following ethical issues related to the randomisation process were explored: fairness and equality, transparency and accountability. Respondents were stakeholders who took part in the randomisation process in the RISE project or traditional leaders in the same communities. Participants were sampled from all the three arms in the study.

### Study settings

One district from each province was purposively selected namely, Mazabuka (Southern province) and Kapiri Mposhi (Central province). Data was collected from three schools in each district: one control, one economic intervention and one combined intervention school, that is, 6 schools in total.

### Sampling

Purposive sampling was used, targeting people that participated in the randomisation process: the head teacher and PTA representative for each selected school. In addition, the representative from the Ministry of Education who had been present in the same ceremony was also interviewed. One headman from each district was interviewed to explore community perceptions among those who had not been present during the ceremonies. A total number of 16 respondents were interviewed. Saturation was reached before sampling was complete.

### Data collection

In this study, face-to-face interviews were done from 22nd May to 7th June, 2018, 2 years after the public randomisation ceremonies in the RISE project. One-to-one interviews helped to capture rich information on individual perceptions and experiences. Interviews were recorded using voice recorders with permission from participants. Information sheets and consent forms were in English and two local languages, Bemba and Tonga. The language used depended on individual participants’ preferences, and interviews were thus conducted in English and Bemba in both districts. The first author conducted all the interviews. A semi-structured interview guide was developed. In Table 1 are some of the main questions asked during the interviews. The interviews started with open-ended questions, and the few closed questions were followed by probing and paraphrasing in order get respondents’ point of view and understanding. This interview guide was flexible, fluid and no fixed steps were applied at any point of the interviews.

**Table 1:** Main questions asked during the interview

Understanding	
1. What do you remember from the randomisation ceremony of the RISE project, conducted in July 2016?	
2. Please tell me what happened at the randomisation ceremony for xx and yy districts	
3. Can you tell me what information you received about the randomisation before the ceremony?	
4. Who participated in the randomisation ceremony together with you from xx school?	
Perceptions	
1. Do you think the randomisation was done in a transparent manner? Why/ why not?	
2. Was the information given during the ceremony about the randomisation process clear? Were you given enough time to seek clarifications?	
3. How fair do you think the randomisation for the RISE project was?	
4. How satisfied are you with the support package that your school was assigned to?	
5. Is there anything that should have been done differently during the randomisation process?	
6. How satisfied are other community members with the support package that your school was assigned to?	
7. How fair do people in your community think the randomisation for the RISE project was?	

Interviews were done in school offices or where the respondents felt was convenient. A poster written ‘interviews in progress’ was stuck outside the office door in order to avoid people entering the office whilst interviews were taking place since this could jeopardize the voice recording process. Each interview lasted about 50 to 60 min.

### Data analysis

Data analysis is a process of bringing order, structure and meaning to the mass of collected data [30]. Our study applied a qualitative, explorative approach. Qualitative research is flexible and unique. It is fluid in nature and evolves throughout the research process. The data collection tool was an interview guide, which was flexible, and no fixed steps were followed. New questions, paraphrasing, and probing was applied in order to come to a deeper understanding of the phenomenona without diverting from the objectives of the study [30].

The content of the data from interviews was explored at the end of each working day. The researchers reflected on the emerging materials, and adjusted the interview guides to enhance their relevance in subsequent days. Transcription and translation were done simultaneously with attention being paid to preserving the original meanings, including culturally embedded content. The interviews were supplemented by hand written field notes. After transcribing the content verbatim, we familiarized ourselves with the data by reading through several times to understand the details. Data was analyzed manually. We applied a bottom up approach starting with descriptive codes that we applied directly to data. We then gathered codes into conceptual categories which had similar characteristics for the purpose of data grouping. The categories were later summarized into interpretive themes. Themes and connections were used to explain the findings as well as attach meaning and significance to the analysis. The first author shared the interviews and codes with other authors in the study. Discussions of the codes were done by all authors to come up with themes.

### Ethical considerations

This study is part of the ongoing RISE study approved by the University of Zambia Biomedical Research Ethics Committee (UNZABREC) on 7th September, 2015 (ethics reference number is: 021–06-15) and the Regional Ethics Committee of Western Norway (REK-Vest).

The purpose and procedures were explained to the stakeholders before consenting. They were given enough time to assimilate the information that was given to them. All the 16 respondents who were approached agreed to take part in the study. Respondents were informed that they could withdraw from the study at any point in time and that this would have no effect in an event that their child was part of RISE. All interviews were conducted in privacy and strict confidentiality was kept.

Respondents’ names were not connected to the audio files of interviews in any way. After the audio recordings were transcribed, recordings were destroyed to avoid voice identification. There were no direct and immediate benefits for the study respondents. However, the findings can inform other community-based RCTs of what aspects of the randomisation that may be particularly difficult for lay people to understand. This will provide best practices and help strengthen the informed consent procedures of RCTs.

## Results

A total number of 16 respondents participated in this study. Two were females; and 14 were males. They were aged between 30 to 50 years. Given that 8 respondents were headmasters/teachers and DEBS officials and college education is a prerequisite for holding these positions, we know that these participants had at least a certificate or degree. The rest of the respondents’ educational background is unknown.

The main themes identified related to understanding the randomisation were problems distinguishing research from a programme and having a “good hand”. The sub-themes were luck and spirituality. There were two main themes related to perceptions of the randomisation process: procedural and substantive fairness. Sub-themes for procedural fairness were transparency, equal chance, voluntarism and fairness. The sub-theme for substantive fairness was unfairness.

Table 2 below shows the overview of themes.

**Table 2:** Overview of themes

	Theme	Sub-theme
Understanding of randomisation	1. Problems distinguishing research from a programme 2. Having a “good hand”	1. a) Luck b) Spirituality
Perception of randomisation process	1. Perception of procedural fairness	1. Transparency 2. Equal chance and voluntarism 3. Fairness
	2. Perception of substantive fairness	1. Unfairness

### Problems distinguishing research from a programme

During the community engagement in 2016, respondents had questions, and they expressed that their questions were answered satisfactorily. However, the respondents’ understanding of the purpose of randomisation were varied. Some respondents that understood randomisation was conducted for research purposes, but most of them did not. Some thought the “lottery” was conducted to allocate aid, not for research purposes, and they had trouble distinguishing research from aid.

“*It was going to be fair if they allocated all groups in three. Even us we felt for them for we were saying that we came together in this hall, it was to be fair if they all received group three.” (Respondent 1, group 3)*

Respondent’s questions were generally focused on the design of the intervention packages and on the issue of packages being unequal. They had this to say:

*“We had questions like, why have you chosen to give this group this much and not the other group? instead of giving all of them equal package. How did you pick the schools? Because there are many schools. How did you differentiate these schools into categories? What did you consider?”* (Respondent 1, group 3)

*“He did mention that there was going to be a raffle where schools will be put in categories. Even if we went there we knew that there was this issue that was going to take place, but to which category we were going to belong to, we did not know”.* (Respondent 1, group 2)

Some respondents did not understand that the randomisation process would only be conducted once. They were hoping to receive a different or better package when the RISE project would conduct another “lottery”:

*“I know that one day RISE may come back to us because they should have a record that we picked this group one. Of course they cannot give the same group again. You cannot be given the same nshima you ate at lunch at supper time”.* (Respondent 9, group 1)

Another echoed:

*“Even when RISE comes for the second round, they will do a raffle and what of if we pick the same group again, what will happen to us? It will not be good to our side. My only hope is that the next randomisation will not disadvantage the already disadvantaged schools like my school”. (*Respondent 11, group 1*)*

### Having a “good hand”

Another understanding of the randomisation was that it was influenced by luck and spirituality. Some respondents believed that the outcome was influenced by their prayers to God. They associated the allocation to package two or three to being lucky and prayerful. They perceived those who went to represent them at the ceremony to be people who are blessed, and that they had “good hands”, that is had good fortune.

*“We were praying hard that we either fall in group two or three, not group one. If we had our own way, we would have loved to be in group three*. *They were saying MM (name of person) we thank you. God has answered our prayers. They said MM, you are a lucky person, you are blessed. Had it not been for you, we wouldn’t have been in category two. And most of the parents are happy with me”*. (Respondent 4, group 2)

### Perception of procedural fairness

#### Transparency

Generally, all respondents mentioned that the randomisation process was transparent. Through their observations, they perceived the process as being open, clear, and people who were conducting the process were available, approachable and quick to respond to their questions***.*** There was no account about conflict of interest, and voluntary participation by the chiefs was observed. The process was perceived to be unbiased and hence nobody mentioned suspicions of manipulation. Being able to witness the whole process was another aspect that people were happy with.

*“It was transparent and open. To me if that could be the way we can be voting even in political parties, it can be fair. That was so open, we were different people from different groups”.* (Respondent 7, group 1)

*R: “…. But I want you to go into details why you think it was transparent”.*

*P: The way it was just conducted; everyone was carried on board from one activity to another. For example, when the box was first shown, from where the papers to be picked to everybody else, there was nothing in the box”.* (Respondent 7, group 1)

*“… there was nobody who told that you go and pick that one. Or to say, come here. Do this so that you get that. I never saw that. What was on the papers is what each school got. There was no secrecy involved in that. It was done in the open”.* (Respondent 14, group 2)

The fact that chiefs and /or their representatives were part of the process reassured the respondents as they were regarded to be responsible and accountable.

*“It was very transparent and fair. The people who were picking the numbers were the chiefs. We all saw what was happening. Can a chief take bribes against his own people?”* (Respondent 13, group 2)

They also mentioned that they did not know the people who were conducting the process, but they knew they were RISE project staff members from University of Zambia and from Norway. The presence of community leaders and RISE project members gave a sense of trust among respondents.

*“Even the people who were conducting the program, we did not know them. No one could say because I know this one, let me do this. It was so difficult to connive with any person because people came from different places”.* (Respondent 7, group 1)

#### Equal chance

The respondents did not perceive that the allocation had been predetermined in any way. *“There was no pointing that such and such a school you belong to this group. It was the papers that guided the groups”.* (Respondent 2, group 3).

#### Fairness

Fairness was perceived and experienced in different ways, depending on a person’s understanding of what was going on. Some respondents perceived the process as fair because it was conducted in a transparent manner. Some respondents indicated it was fair because people who picked the numbers were impartial and could not be manipulated. Some of the respondents used the concepts of transparency and fairness interchangeably. According to them, fairness depended on transparency.

*“It was fair and transparent. Those people who made the groups, to me there was some intelligence in it. Why I say so is nothing which I could ask anybody to say why have you done so because those groups that were put there and the numbers was clear. There was some intelligence so that there was no corruption. If it was not done like that, I was going to do corruption because I am a human being. I could have pulled some officer in the corner and bribed them to give me group three, but there was transparency, it tied up everything and made it to be fair and transparent.”* (Respondent 7, group 1).

During the interviews, respondents mentioned that they explained the randomisation process to the community members since they did not attend the ceremony. Respondents were asked if community members were satisfied with the outcome of the randomisation process. Those from group one said that some community members perceived the process as unfair. It could be that the process was not clearly understood because they did not attend the ceremony. Other community members, after being explained to, came to understand and were satisfied with the package they received. Some respondents mentioned that the way RISE conducted the randomisation process itself was professional. They did not think of any better way of performing the randomisation and were satisfied.

*“They were satisfied with the process that it was fair and transparent. But what they did not like were the groups. They would have loved to be in the other group. Maybe they were looking at the support the school was to receive. And looking at some schools, we seem to have more vulnerable children so they thought if their school could be in group three maybe then children could have benefitted. The unfairness was in the packaging”.* (Respondent *12, DEBS representative).*

A few indicated that it was fair in the sense that the RISE project is a research project and it was done according to their plan. Others indicated that it was fair because whatever RISE promised, they came to fulfil.

*“What we were told is exactly what happened. RISE said they will do so and so and they did exactly as promised. So it was fair”.* (Respondent 13, group 2)

### Perceptions of substantive fairness

There were some respondents, especially those from group one, who indicated that they were not happy with the benefits they received even if the process itself was transparent and fair. During the interviews, some of the respondents expressed strong feelings/emotions of disappointment and were disturbed and discouraged because of the package their schools received. They felt that it had not been worthwhile attending the randomisation ceremony.

*“It was not a happy moment for us. And that was how even us who are crying lost”*. (Respondent 11, group 1)

The respondents perceived and understood fairness in relation to the contents of each package. They perceived group one (control) as a “bad” group because only material support was provided. Group two (economic intervention) was perceived as a “better” group as compared to group one and group three as the “best” group because it was comprehensive. They complained that the support packages were unfair because the gap between the support provided to the different arms was huge. Generally, respondents wished and hoped to be in category three. One respondent whose school fell under group one complained bitterly that the group allocation was not done fairly. Some participants mentioned that the RISE project segregated schools by not allocating all schools under group three and allowing other pupils to benefit more than others. They emphasized that the RISE project should not have conducted a raffle. One respondent alluded:

*“Group one was done unfairly. To me, it was too much for group three. It was going to be fair if they shared with these other groups for balancing purposes. But to me they were supposed to share, not equally but at least to balance up so that each group benefits in all areas”.* (Respondent 7, group 1)

Another echoed:

*“The package allocation of these categories was also unfair. This is because some schools or some students in certain schools benefited more than the other pupils in some other schools. That also showed some element of segregation in the whole project”.* (Respondent 1, group 3)

Some respondents accused the RISE project to have further disadvantaged already vulnerable schools and explained that the RISE project should have considered the locality and environment of the schools before allocating packages. Some communities are located in remote areas and most of the parents are poor. They indicated that some schools that ended up in group three were located in non-remote areas and had parents employed in government and other parastatal organizations. They felt that the RISE project should have favored those who were socio-economically disadvantaged such as those in remote areas and peasant farmers.

*“I still stand my ground that it wasn’t fairly done. RISE project should have actually considered the location of schools. If we compare XX with YY (names of localities), most of the pupils that go to this school are coming from ZZ and UU (names of localities). There are fewer pupils that are vulnerable in these places. When you look at this locality, most of the pupils are vulnerable because their parents are farmers who are subsistence farmers, not commercial farmers”.* (Respondent 4, group 2)

A couple of the respondents insinuated that the project had contributed to some conflict and social disharmony between the schools.

*“Others felt disappointed because their schools fell in group one. PP (name of school) fell in group one because that head teacher complaining that for us it is just materials. And I was very quick to answer him that at least you are in town and most of the parents are in employment and if they are not in employment, they are at least doing something.”* (Respondent 4, group 2)

Another respondent echoed this and expressed that conducting a “lottery” in the first place was not a good idea.

*“They should not conduct the raffle. Because if they conduct the raffle again, you may not know, you may pick the same group again. In the end, you may find that certain school may not even welcome RISE”.”* (Respondent 11, group 1)

During the discussions about fairness, the respondents also mentioned that the project had contributed to social disharmony within the communities because of the eligibility criteria, which excluded boys and girls in other grades. Although this was not related to the randomisation as such, it contributed to perceptions that RISE was unfair:

*“The boys also want money like girls. They go out to look for jobs at young age. There are some who also marry at a young age and they stop school. This is because the parents do not have money to support him in school. In this country, we are struggling because of money problems. You can find that during holidays, children remain in school for tuitions. Those who remain in school are the children whose parents have money to pay for extra tuitions, say, twenty kwacha. Those without money can’t attend extra lessons and it means that they lag behind. Here girls are vulnerable but boys also are also vulnerable. Fairness must be applied. We need balance”* (Respondent 13, group 2)

## Discussion

The understanding of the randomisation process was varied among the respondents who had been present during the randomisation ceremonies. Everyone was happy with the process being transparent and fair. However, misunderstandings regarding the rationale behind the randomisation process resulted in some respondents perceiving the randomisation as unfair because they thought the purpose was to allocate aid. They did not understand the distinction between research and aid. The majority had concerns that a “lottery” should not have been used, but the benefits should have been allocated based on need. Their perception of whether the allocation was fair was influenced by which support package their school was allocated to.

Some of the respondents expressed that they thought randomisation was a bad idea because it did not ensure equal distribution of benefits, clearly indicating that they had not understood the purpose of the randomisation. The information the respondents had been given appeared to have been insufficient for them to understand that randomisation was necessary to ensure that the study arms were comparable and that allocating the benefit packages based on need would undermine the scientific validity of the study. Although the term ‘lottery’ was used to depict and explain randomisation, this did not help. The interviews indicate that the public ceremonies had not achieved the objective of creating an understanding for why randomisation is important in an RCT. This was probably partly because many community members also struggled to understand what research is although they were well informed during the community engagement about the RISE project being a research study. Considering that the support packages that were to be tested resembled support provided by certain non-governmental organizations (NGOs), the RISE project may have been more prone than many other RCTs to be misunderstood as an aid project, despite repeated efforts to explain the purpose of the project and that the implementers were research institutions. This indicates that more preparations should have been made to explore how best to explain these key concepts in a rural Zambian setting.

Judging from the examples they gave, the respondents appeared to understand the central aspects of transparency. The respondents did not use the concept accountability directly themselves, but the way many of them talked about the role of the chiefs in the ceremonies, indicated that they perceived the chiefs to be accountable superiors who did not have the interests of only one community in mind. It appears that any suspicions about manipulation were prevented by the involvement of accountable persons and the use transparent procedures during the public ceremonies.

The respondents had different understandings of the concept of fairness. A few used the term in unusual ways, for example to indicate that they they were satisfied with the outcome. The majority focused on substantive fairness, which to them implied that everyone should receive the same benefits or the benefits should be proportionate to people’s needs. A few of the respondents also used the term fairness in relation to the procedural aspects and argued that the process was fair because it was transparent and was not biased. Our findings are thus in line with those of Stone [33] who found that the distribution of goods across individuals or groups raises questions of justice. Several other studies have found that allocation of interventions may potentially exacerbate inequalities among groups of people and this can disturb communities, creating social disharmony [6, 13, 14, 32, 34, 39]. In this case respondents’ perceptions and experiences brought in anger towards other communities. However, during the interviews the respondents also mentioned several examples of how the restrictive eligibility criteria created even deeper resentment within the community as community members found it unfair that boys and girls in other grades were excluded from the trial. If the randomisation units had been individuals instead of clusters, it is thus possible that the social disharmony brought by the randomisation could have been even stronger than what we found when all the participants in the same community were allocated to the same arm.

Haushofer and Shapiro [13] found that people generally judge randomisation as unacceptable when one treatment is better than the other. Scientifically it is also regarded as unethical to conduct an RCT if there is no genuine uncertainty in the expert community over whether an intervention will be beneficial (called the principle of equipoise). The respondents in this study unanimously thought that the combined support package was better than the other packages, even though previous research on similar intervention components have found contrasting effects in different settings [2, 24]. This indicates that more information on findings from previous studies of economic support and community dialogue should have been included in the sensitization meetings in order to reduce the disappointment in the communities allocated to the control and the economic support arms.

This study added useful information to the body of knowledge on the popular understanding of randomisation. However, the study experienced the following challenges: interviews were conducted 2 years after the randomisation process and some respondents did not attend the randomisation ceremonies. Thus some of the respondents may have forgotten or never had a good understanding of the process. This means that some of the current misunderstanding of the randomisation process could have been due to not remembering what happened, but we have no way of telling the difference. Since we did not collect information on the educational background of the respondents, we cannot rule out that their understanding of the randomisation procedures were influenced by their educational level. We did not collect information from community members who had not been present during the randomisation ceremonies, only indirect information on community members’ views from interview respondents who represented the community, and thus we cannot ascertain how widespread the misunderstanding and the perceptions of unfairness were. We recommend that future studies which explore how randomisation is understood in cluster randomized trials also include perceptions and experiences of trial participants themselves.

## Conclusion

The public randomisation ceremonies did not succeed in creating an understanding of the purpose of randomisation in a RCT among most of the respondents. However, the procedures of the public ceremonies were generally perceived as fair and transparent, and the fact that traditional leaders were part of the process reassured the respondents as they regarded them as impartial and accountable. For substantive fairness, people thought that the purpose of the “lottery” was to allocate aid and that the allocation should have been based on need rather than chance. The provision of clear and accurate information to participants about RCTs is important but this alone may not ensure consistent interpretation of core concepts such as randomisation or “lottery” and research. Thus formative research on how to better explain the concept of randomisation in lay language in the context where a RCT is planned, can be useful to ensure that the community engagement process helps research participants understand what to expect and do not withdraw because they think they have been unfairly treated.

## Data Availability

The datasets during and/or analysed during the current study are available from corresponding author on reasonable request.

## Abbreviations

CRCTs: Cluster Randomised Controlled Trials
CSO: Central Statistical Office
DEBS: District Education Board Secretary
DHHS: Department of Health and Human Services
MRC: Medical Research Council
PTA: Parents Teachers Association
RCTs: Randomised Controlled Trials
RISE: Research Initiative to Support the Empowerment of Girls
UNFPA: United Nations Fund for Population Activities
WHO: World Health Organisation
